# A prospective multicenter study of treosulfan in elderly patients with recurrent ovarian cancer: results of a planned safety analysis

**DOI:** 10.1007/s00432-012-1221-3

**Published:** 2012-04-15

**Authors:** Sven Mahner, Gülten Oskay-Özcelik, Elke Heidrich-Lorsbach, Stefan Fuxius, Harald Sommer, Peter Klare, Antje Belau, Birgit Ruhmland, Thomas Heuser, Heinz Kölbl, Susanne Markmann, Jalid Sehouli

**Affiliations:** 1 Department of Gynecology and Gynecologic Oncology, University Medical Center Hamburg-Eppendorf, Hamburg, Germany; 2 Department of Gynecology and Gynecologic Oncology, Charité-University Medical Center Berlin, Berlin, Germany; 3 Alcedis GmbH, Giessen, Germany; 4 Private Practice Oncology, Heidelberg, Germany; 5 Department of Gynecology, University of Munich, Munich, Germany; 6 Private Practice Gynecology and Gynecologic Oncology, Berlin, Germany; 7 Department of Gynecology and Gynecologic Oncology, University Greifswald, Greifswald, Germany; 8 Private Practice, Berlin, Germany; 9 Herford Clinic, Herford, Germany; 10 Department of Obstetrics and Gynecology, University of Mainz, Mainz, Germany; 11 Department of Gynecology, University of Rostock, Rostock, Germany

**Keywords:** Ovarian cancer, Elderly, Chemotherapy, Patient preference, Safety, Treosulfan

## Abstract

**Background:**

Treosulfan, an alkylating agent, has demonstrated activity in recurrent ovarian carcinoma. It is equieffective as oral (p.o.) and intravenous (i.v.) formulation. To explore the preference and compliance of elderly patients regarding p.o. or i.v. treosulfan for the treatment of relapsed ovarian carcinoma, women aged 65 years or older were included in this prospective multicenter study. Since elderly patients usually have several concomitant diseases and experience more treatment toxicity, an interim safety analysis was planned and performed after 25 patients finished therapy to assess the tolerability of the treatment regimens.

**Methods:**

Patients had a free choice of treosulfan i.v. (7,000 mg/m^2^ day 1 of a 28-day cycle) or p.o. (600 mg/m^2^ day 1–28 of a 56-day cycle) for a maximum of 12 cycles (i.v.) or 12 months (p.o.). Indecisive patients were randomized. Toxicity was evaluated according to the NCI-CTC version 2.0.

**Results:**

Twenty-five of 51 recruited patients completed therapy at the time of the planned interim analysis (median age, 75 years; range, 70–82). Median ECOG was 1, and median number of prior chemotherapy regimens was 2. A median number of 4 cycles (range, 1–12) were administered per patient. Anemia was the most common hematological toxicity (88 % of patients). Most frequent non-hematological toxicities were nausea (76 %), constipation (68 %), and fatigue (64 %).

**Conclusion:**

Treatment was generally well tolerated despite the fact that most patients suffered from multiple comorbidities and were heavily pretreated. There were no unexpected hematological or non-hematological toxicities. Based on this safety analysis, the next step of study recruitment was continued.

## Introduction

In Europe, 58 % of all cancers occur in women older than 65 years with a peak in the 7th and 8th decade (Pignata and Vermorken 2004). Median age at diagnosis of ovarian cancer is 63 years (Altekruse et al. 2010). As the proportion of older adults in the population is growing, the number of women with ovarian cancer is expected to increase worldwide. Irrespective of these facts, older women are underrepresented in clinical trials, mainly due to the common misbelieve that elderly patients would not tolerate treatment toxicities. Restrictive inclusion criteria regarding age, adequate hematological, renal, and cardiac functions lead to non-enrollment of elderly patients in clinical trials (Harter et al. 2005). However, previous research could demonstrate that the decision to treat elderly patients with chemotherapy should be based on patients’ functional age rather than chronical age (Pallis et al. 2010a, b). Therefore, our trial also included a multidimensional geriatric assessment of each enrolled patient.

Treosulfan is the prodrug of a bifunctional alkylating cytostatic agent that is active in ovarian carcinoma and other solid tumors (Gropp et al. 1998; Köpf-Maier and Sass 1996). It can be administered oral or as intravenous infusion (Hilger et al. 2000) and is known for its relatively low hematological and non-hematological toxicity.

In the present study, women with relapsed ovarian cancer aged 65 years or older were enrolled after failure of platinum-containing therapy. Patients had free choice between treosulfan p.o. and i.v. Since elderly patients usually suffer from several concomitant diseases and experience more treatment toxicity, an interim safety analysis was planned and performed after 25 patients finished therapy to assess the safety and tolerability of the treatment regimens.

## Patients and methods

This open-label multicenter phase-IIIb trial was conducted at 47 German institutions; the first 25 patients analyzed in this safety analysis were recruited in 10 centers.

Primary aim of the study was to explore the preference and compliance of elderly patients for p.o. or i.v. treosulfan. Toxicities, progression-free survival, overall survival, geriatric assessment (iADL—*instrumental activities of daily living* and ADL scale), and quality of life were defined as secondary objectives. Toxicity analysis focused on hematological toxicities as well as on number of patients with treatment delay or discontinuation. There were no predefined toxicity limits specified in the study protocol concerning continuation of the study, which was subject to regular review of the data safety monitoring board.

The study was performed according to ICH-GCP (International Conference on Harmonisation-Good Clinical Practice) guidelines. An independent monitoring institute was responsible for data control (Alcedis GmbH, Giessen, Germany). Approval from local ethical committees was gained at every participating center, and written informed consent was provided by each participant.

Patients with histologically confirmed recurrent ovarian cancer having received at least one prior platinum-based cancer therapy were allocated to this trial. The number of prior therapies was initially planned as 1 and subsequently changed by amendment to at least 2 previous therapies after 7 patients had been included. The amendment was implemented to reflect the new treatment standards of topotecan or liposomal doxorubicin as treatment of choice for platinum-resistant ovarian cancer at second line.

Minimal age for inclusion was 65 years with no upper limit. Eligible patients were required to have a life expectancy of more than 3 months, and an Eastern Cooperative Oncology Group (ECOG) performance status less than 3. Two-dimensional measurement of the tumor or, alternatively, increase in CA125 values greater than 100 U/ml were necessary.

Serum creatinine levels and total bilirubin had to be below 1.25 times the upper limit of normal, in case of liver metastases bilirubin was allowed to be up to 5 times upper limit of normal. Adequate bone marrow function as indicated by a leukocyte count greater than 2,000/μl and platelet count greater than 100,000/μl was required.

Patients suffering from secondary malignancies that influenced the prognosis were not eligible. Current treatment with any chemo-, immuno-, or hormonal therapy as well as previous treatment with treosulfan was additional exclusion criterion.

Before start of therapy, registered patients could choose between oral and intravenous treosulfan. Indecisive women were randomized to one of the treatment arms. The patients’ preference regarding the way of application will be part of the final analysis of this trial. Oral medication was given as 600 mg/m^2^ treosulfan on day 1–28 of a 56-day cycle.

In the i.v. group, patients received 7,000 mg/m^2^ over 15–30 min on day 1 of a 28-day cycle. No general premedication was defined, and participating centers could apply their specific standards. Primary supportive administration of granulocyte-colony-stimulating factor (G-CSF) was allowed. Concomitant use of food, especially milk, was recommended for all women receiving treosulfan as oral application.

To account for the limited hematopoietic resources of elderly patients, chemotherapy was applied only if leukocyte count was ≥3.5 × 10^9^/l and platelet count was greater than 100 × 10^9^/l.

Dose reductions were applied in case of severe thrombocytopenia or leucopenia. No re-escalation of a reduced dose was allowed. Progress of the tumor, intolerable grade 3 or 4 toxicities (excluding nausea grade 3), and/or a treatment delay exceeding more than 2 weeks lead to discontinuation of treatment.

Blood samples for hematology (hemoglobin, leukocytes, and platelets) and blood chemistry (creatinine, alkaline phosphatase, SGOT, SGPT, gamma-GT, LDH, total bilirubin, albumin, and total serum protein) as well as CA-125 were collected prior to each cycle.

Toxicity was graded according to the classification of the National Cancer Institute “Common Terminology Criteria for Adverse Events” (CTCAE) in the version of 1999 (version 2.0) at the end of every cycle. Safety was assessed by the analysis of toxicity parameters (occurrence of adverse events, serious adverse events, and deaths).

The EORTC QLQ-OV-28-questionnaire was used to assess the quality of life. Patients were asked to fill in the questionnaire before start of therapy, during and at the end of treatment. Comorbidity was determined according to the “Cumulative Illness Rating Scale” at the beginning and the end of study therapy. For the geriatric assessment, the Scales of Lawton and Brody were used. Patients were asked for self-concept regarding the instrumental activities of daily living at registration, during therapy and at the end of study treatment. If a patient reached <8 points in the iADL, the Barthel ADL Index was used additionally.

## Results

This safety analysis was performed after 25 patients with recurrent ovarian cancer were included in this study and finished treatment. Baseline characteristics are shown in Table 1. Median age was 75 years (range, 70–82). The majority of patients had advanced stage disease (FIGO III/IV) at initial diagnosis. Forty-four percent of the patients had a recurrence-free interval after primary therapy of more than 12 months. All patients had prior surgery and a median of 2 chemotherapies. Eight patients (32 %) underwent second-line and seven patients (28 %) third-line therapy. Two patients had more than 4 recurrences before inclusion into the study. Two patients had received hormone therapy before inclusion. Distant metastases were rare and localized to the liver or lung. Nineteen patients had an ECOG performance status of 0 or 1, and six patients had an ECOG of 2.

**Table 1:** Patient characteristics

No. of patients in study	51
No. assessed for toxicity	25
Median age, in years (range)	75 (70–82)
ECOG^a^	
0	2 (8.0 %)
1	17 (68.0 %)
2	6 (24.0 %)
FIGO-stage at initial diagnosis	
IIA	2 (8.0 %)
IIC	1 (4.0 %)
IIIA	1 (4.0 %)
IIIB	2 (8.0 %)
IIIC	15 (60.0 %)
IV	4 (16.0 %)
Grading at initial diagnosis	
G2	10 (40.0 %)
G3	11 (44.0 %)
GX	4 (16.0 %)
Histology at initial diagnosis	
Serous papillary	19 (76 %)
Others or NOS	6 (24 %)
Relapse-free interval after primary therapy	
<6 months	5 (20.0 %)
6–12 months	9 (36.0 %)
>12 months	11 (44.0 %)
Previous therapies	
Surgery	25 (100.0 %)
Radiotherapy	0 (0.0 %)
Chemotherapy	25 (10.0 %)
Number of previous chemotherapies, median (range)	2 (1–6)
1	6 (24.0 %)
2	8 (32.0 %)
3	7 (28.0 %)
4	2 (8.0 %)
5	1 (4.0 %)
6	1 (4.0 %)
Hormone therapy	2 (8.0 %)
Therapy situation at study entry	
1st relapse	7 (28.0 %)
2nd relapse	9 (36.0 %)
3rd relapse	5 (20.0 %)
4th relapse	1 (4.0 %)
>4th relapse	3 (12.0 %)
Location of distant metastases	
Liver	6 (24.0 %)
Lung	2 (8.0 %)
Concomitant diseases, median number (range)	5 (1–9)

^a^Eastern Cooperative Oncology Group Performance Score

The median number of concomitant diseases was 5, with a range from 1 to 9, mostly cardiovascular or orthopedic (Table 2).

**Table 2:** Concomitant diseases

Site	*n*	% (*n* = 122)
Blood pressure	18	14.8
Musculoskeletal system	18	14.8
Heart	13	10.7
Lower gastrointestinal tract	11	9.0
Blood vessels	10	8.2
Neurological disease	9	7.4
Metabolism and endocrine system	8	6.6
Respiratory system	9	7.4
Liver	7	5.7
Kidney	7	5.7
Upper gastrointestinal tract	6	4.9
Urinary tract	3	2.5
Psychiatric	3	2.5
	122	100.0

The majority of the patients (*n* = 23) decided on the application form, 2 patients were indecisive and allocated to a treatment arm by randomization. Altogether 22 patients (2 by randomization) received i.v. application, and 3 patients decided to take treosulfan orally. Patients received a median of 4 cycles of study medication (range, 1–12). The median scheduled dose per cycle of 7,000 mg/m^2^ (i.v.) and 600 mg/m^2^ (p.o.) per day could be applied (Table 3). Median total cumulative dose of the p.o.-patients was 70,000 mg, whereas patients getting the i.v. application had a median total cumulative dose of 49,840 mg.

**Table 3:** Treatment delivery

Total cycles (patients)	117 (25)
Cycles per patient, median (range)	4 (1–12)
Cycles with treosulfan i.v. (patients)	108 (22)
Cycles with treosulfan p.o. (patients)	9 (3)
Treosulfan dose i.v. (mg/m^2^), median (range)	7,000 (4,060–7,000)
Treosulfan dose p.o. (mg/m^2^) per day, median (range)	600 (600–600)
Dose reduction i.v., cycles/patients	9 (8.3 %)/2 (8.0 %)
Dose reduction p.o., cycles/patients	0 (0 %)/0 (0 %)
Cycles delayed (p.o. and i.v.)	20 (17 %)

With oral therapy, no dose reductions were necessary, whereas two patients in the i.v. therapy arm received 9 cycles (8.3 %) with reduced treosulfan dose. Overall, 17 % of cycles were delayed. One woman received the maximum planned number of 12 cycles, 24 patients stopped therapy early. The reasons for therapy discontinuation are shown in Table 4. In most cases, the reason was progressive disease (48 %). Two patients died during the study period.

**Table 4:** Reasons for early therapy discontinuation [less than 12 cycles (i.v.) or 12 months of therapy (p.o.)]

Reasons for early discontinuation of therapy	Number of patients	% Patients (*n* = 24)
Progressive disease	12	48.0
Patients choice	3	12.0
Other reasons	3	12.0
Death	2	8.0
Hematological toxicity of grade 3 or 4	2	8.0
Non-hematological toxicity of grade 3 or 4	1	4.0
Relevant concomitant disease	1	4.0

All non-hematological and hematological toxicities occurring during therapy are shown in Tables 5 and 6. The majority of all documented toxicities were of grade 1 or 2. Nausea and constipation were the most frequently recorded non-hematological toxicities. Altogether 21 non-hematological toxicities of grade 3 and 1 non-hematological toxicity (neuropathy) of grade 4 occurred (Table 5).

**Table 5:** Non-hematological toxicities: highest grade per patient (in alphabetic order)

Non-hematological toxicities	Grade 1 (*n* = 25)	Grade 2 (*n* = 25)	Grade 3 (*n* = 25)	Grade 4 (*n* = 25)	Grade unknown (*n* = 25)	
	Patients (%)	Patients (%)	Patients (%)	Patients (%)	Patients (%)	
Abdominal pain or convulsion	5 (20.0 %)	3 (12.0 %)	1 (4.0 %)			9
Acute viral rhinopharyngitis	1 (4.0 %)					1
Alopecia	4 (16.0 %)	1 (4.0 %)			2 (8.0 %)	7
Amnesia			1 (4.0 %)			1
Anorexia	3 (12.0 %)	1 (4.0 %)				4
Arthralgia	1 (4.0 %)	3 (12.0 %)				4
Arthritis	1 (4.0 %)		1 (4.0 %)			2
Ascites			1 (4.0 %)			1
Back pain		2 (8.0 %)				2
Chest pain (not cardial and not pleuritic)		1 (4.0 %)	1 (4.0 %)			2
Common cold	1 (4.0 %)	1 (4.0 %)				2
Conjunctivitis	1 (4.0 %)	1 (4.0 %)				2
Constipation	7 (28.0 %)	5 (20.0 %)	2 (8.0 %)		3 (12.0 %)	17
Diaphoresis	1 (4.0 %)					1
Diarrhea	1 (4.0 %)	1 (4.0 %)	1 (4.0 %)		3 (12.0 %)	6
Dyspnea	2 (8.0 %)	2 (8.0 %)				4
Dysuria		1 (4.0 %)				1
Edema	2 (8.0 %)	2 (8.0 %)				4
Fatigue	12 (48.0 %)	2 (8.0 %)	2 (8.0 %)			16
Gastritis		1 (4.0 %)				1
Headache	2 (8.0 %)					2
Hearloss	1 (4.0 %)					1
Hemorrhage	1 (4.0 %)					1
Hoarseness	2 (8.0 %)					2
Hydronephrosis	1 (4.0 %)					1
Hydronephrosis		1 (4.0 %)				1
Hyperkalemia			1 (4.0 %)			1
Hypoglycemia			1 (4.0 %)			1
Hypotension	1 (4.0 %)					1
Ileus			2 (8.0 %)			2
Incontinence			1 (4.0 %)			1
Infection without neutropenia	1 (4.0 %)		1 (4.0 %)			2
Insomnia	1 (4.0 %)					1
Myalgia	1 (4.0 %)	1 (4.0 %)				2
Nausea	12 (48.0 %)	5 (20.0 %)	1 (4.0 %)		2 (8.0 %)	20
Pain (bone)	3 (12.0 %)	2 (8.0 %)				5
Peripheral arterial occlusive disease			1 (4.0 %)			1
Peripheral neuropathy	8 (32.0 %)	3 (12.0 %)		1 (4.0 %)		12
Pruritus			1 (4.0 %)			1
Pyrexia (without neutropenia)	1 (4.0 %)	1 (4.0 %)				2
Skin-related toxicities	7 (28.0 %)	2 (8.0 %)			3 (12.0 %)	12
Sleep disorder	1 (4.0 %)	1 (4.0 %)				2
Stomatitis/pharyngitis	5 (20.0 %)	1 (4.0 %)				6
Subileus			1 (4.0 %)			1
Vertigo	2 (8.0 %)	1 (4.0 %)				3
Vomiting	7 (28.0 %)	5 (20.0 %)	1 (4.0 %)		2 (8.0 %)	15
Weight loss		1 (4.0 %)				1
Xerostomia	3 (12.0 %)					3
Sum of events	102	51	21	1	15	190

**Table 6:** Hematological toxicities: highest grade per patient

Hematological toxicities	Grade 1 (*n* = 25)	Grade 2 (*n* = 25)	Grade 3 (*n* = 25)	Grade 4 (*n* = 25)	Grade unknown (*n* = 25)	
	Patients (%)	Patients (%)	Patients (%)	Patients (%)	Patients (%)	
Anemia	9 (36.0 %)	8 (32.0 %)	1 (4.0 %)		4 (16.0 %)	22
Leukopenia	8 (32.0 %)	1 (4.0 %)	3 (12.0 %)		5 (20.0 %)	17
Neutropenia	1 (4.0 %)		1 (4.0 %)	1 (4.0 %)	6 (4.0 %)	9
Thrombocytopenia	10 (40.0 %)	1 (4.0 %)	1 (4.0 %)		4 (16.0 %)	16
Sum of events	28	10	6	1	19	64

The most common hematological toxicities were leucopenia and neutropenia. Leucopenia of grade 3 occurred in 3 patients (12 %) and 1 woman each (4 %) suffered from neutropenia of grade 3 or 4 (Table 6).

Altogether 16 serious adverse events (SAE) were documented for nine patients. The predominant reason for SAE-reporting was hospitalization (14 reports). Death was the matter for the remaining 2 SAEs. An additional patient was hospitalized due to progressive disease and died in hospital. In all 3 cases, death was concerned as not related to study medication and progress of the underlying malignant tumor was stated as cause of death.

## Discussion

Ovarian cancer has the highest incidence in the seventh and eighth decades of life (Gondos et al. 2007; Oleitan et al. 2000). Due to substantial improvements in surgery and chemotherapy regimens for ovarian cancer, longer survival results in increasing number of older patients with recurrent disease. These elderly women are usually less “fit” than younger patients and therefore require specific (mono) therapies with moderate side effects. Combination therapies tend to be more toxic than monotherapies, especially with respect to myelotoxicity (Breidenbach et al. 2003).

The current standard therapy for platinum-sensitive recurrent ovarian cancer (progress 6 months or later after the end of previous therapy) is platinum-based combinations with liposomal doxorubicin, gemcitabine, or paclitaxel (Pujade-Lauraine et al. 2010) and in case of contraindications for platinum the combination of trabectedin and liposomal doxorubicin (Monk et al. 2010). All these combination have a relatively high hematological or non-hematological toxicity in common. For patients with disease recurrence within 6 months (so-called platinum resistance), monotherapies with topotecan, liposomal doxorubicin, paclitaxel, or gemcitabine represent the current standard approach. Again, all three have distinct hematological and non-hematological toxicity (Uziely et al. 1994; Meier et al. 2009; Sehouli et al. 2008). The use of anthracyclines, which are among the most active single agents for the treatment of recurrent ovarian cancer, is often limited in elderly patients due to the high prevalence of cardiac comorbidity.

The management of recurrent ovarian cancer in elderly women remains to be a clinical challenge. The main goals of treatment strategies are to maintain tumor control and quality of life as long as possible drugs of low toxicity. The alkylating agent treosulfan has previously shown activity as monotherapy against recurrent ovarian cancer exhibiting only mild toxicities (Reed et al. 2006; Keldsen et al. 1998). In several European countries, it is approved for palliative therapy alone or in combination in women with advanced ovarian cancer after failure of platinum-containing therapies, and we present the results of the safety analysis of the first prospective study regarding tolerability of palliative chemotherapy in elderly patients with relapsed ovarian cancer.

In a clinical phase-III trial with platinum-refractory ovarian cancer comparing topotecan versus treosulfan, it was demonstrated before that hematological toxicity was significantly less frequent in the treosulfan arm, whereas non-hematological toxicity was similar in both arms (Meier et al. 2009). Stratification according to platinum sensitivity revealed for the stratum “relapse time up to 6 months after primary chemotherapy” that both treatments were equally effective with regard to overall survival, while progression-free survival was favorable in the topotecan arm.

Median age of the patients in the topotecan and treosulfan arm was 58 (range, 30–78) and 59 (27–77) years, respectively, whereas patients in this current study had a median age of 75 years. In contrast to most published series where patients had only one prior chemotherapy (usually platinum/paclitaxel), patients in our study had a median of 2 prior chemotherapies. At study entry, there were 9 patients (36 %) in second relapse and 5 patients (20 %) in third relapse. Three patients (12 %) even had more than 4 prior relapses. Most of the subjects suffered from concomitant cardiovascular disease and orthopedic problems. In median, 5 concomitant diseases were present. Mostly, the concomitant diseases were classified as mild or moderate; in 15 cases, the grading was severe. The higher number of concomitant disease in elderly patient usually goes along with more concomitant medication. Since the concomitant medication also impose distinct toxicity and side effects, it is important to use chemotherapy, which exerts only moderate toxicity in this context. The patients analyzed here took a median of 4 concomitant medications at the time of registration. Comorbidity and co-medication thus directly influence efficacy and tolerability of chemotherapy, and the choice of therapeutic regimens therefore has to carefully balance risks and potential benefits.

In the current study, most of the observed hematological and non-hematological toxicities were of grade 1 or 2. Therapy with treosulfan is well tolerated in this patient group. Grade 3/4-toxicities normally occurred as individual incidents. Three women discontinued therapy due to grade 3/4-toxicities. Despite the difference in median age and prior therapy of the patients, the quantity of observed toxicities grade 3/4 in this trial is comparable with the toxicities in the published phase-III trial (Meier et al. 2009).

In another randomized study comparing treosulfan and carboplatin as monotherapy in older women (median age, 73 years) with advanced ovarian cancer, hematological toxicities again were comparable to our study, although patients included into that study had no prior chemotherapy (Reed et al. 2006). In the treosulfan arm, neutropenia and leucopenia of grade 3/4 were the most frequently occurring hematological toxicities, leucopenia being nearly twice as high as in the carboplatin arm. Leucopenia and neutropenia also are the most often occurring grade 3/4 hematological toxicities in our study.

In summary, the observed toxicities were in the same range as reported in previous studies with significantly younger patients and less comorbidity or with old women having received fewer previous lines of chemotherapy.

There were no unexpected hematological or non-hematological toxicities. Based on this safety analysis, treosulfan proved to be a safe and tolerable therapeutic option in elderly, heavily pretreated patients and the next step of study recruitment was initiated. Of note, the majority of patients in the interim safety population chose i.v. treosulfan over the oral application. Detailed analysis after completion of the trial will hopefully yield new insight into therapy preference and compliance of elderly patients with recurrent ovarian cancer.

## Abbreviations

ADL: Activities of daily living
iADL: Instrumental activities of daily living (after Lawton and Brody)
ICH: International Conference on Harmonisation of technical requirements for registration of pharmaceuticals for human use
GCP: Good clinical practice
i.v.: Intravenous
p.o.: Per oral
SAE: Serious adverse event
